# Digital Solutions Available to Be Used by Informal Caregivers, Contributing to Medication Adherence: A Scoping Review

**DOI:** 10.3390/pharmacy12010020

**Published:** 2024-01-23

**Authors:** Margarida Espírito-Santo, Sancha Santos, Maria Dulce Estêvão

**Affiliations:** 1 School of Health, University of Algarve, 8005-139 Faro, Portugalmestevao@ualg.pt (M.D.E.); 2 Centre for Health Studies and Development (CESUAlg), University of Algarve, 8005-139 Faro, Portugal; 3 Algarve Biomedical Center Research Institute (ABC-RI), 8005-139 Faro, Portugal

**Keywords:** chronic diseases, digital health tools, informal caregivers, medication adherence

## Abstract

Medication adherence is essential for managing chronic diseases and achieving optimal health outcomes. However, this process is often challenging, particularly for patients with complex care needs. Informal caregivers play a pivotal role in supporting medication management, but they may face resource limitations and a lack of necessary support. Digital health tools offer a promising avenue to enhance medication adherence by providing reminders, education, and remote monitoring capabilities. This scoping review aimed to identify and evaluate digital solutions available to informal caregivers for improving medication adherence. A systematic search of PubMed and Web of Science was conducted using relevant keywords. Four studies were included in the review, examining a variety of digital tools including mobile apps, SMS messaging, and wearable devices. These tools demonstrated efficacy in improving medication adherence, managing disease symptoms, and enhancing quality of life for patients and caregivers. Digital health interventions hold the potential to revolutionize medication adherence among chronic disease patients. By empowering informal caregivers, these tools can bridge the gaps in medication management and contribute to better health outcomes. Further research is warranted to optimize the design, implementation, and evaluation of digital interventions for medication adherence.

## 1. Introduction

Treatment adherence may be defined as the extent to which the person’s behavior, regarding taking medication, following a diet, and/or practicing lifestyle changes, corresponds to the recommendations given by a health professional, and may be affected by several factors, such as socioeconomic conditions, and those related to services, health professionals, treatment, disease, and the patient [1]. As well previous experiences within the process of medication use, the patient’s beliefs and cultural context, lack of motivation, and lack of family and/or social support can also compromise adherence [2].

Although it is crucial to control the progress of diseases, the prescription of complex therapeutic regimens by the physician(s), and the lack of communication between them and the patient, can compromise adherence, as there may be scarce explanation about the benefits and possible drugs side effects. Inadequate communication can lead to avoidable medication errors and hospital readmissions. Factors related to the health system, such as the cost of medicines and lack of time during consultations to ask questions about the therapy, are two obstacles in the improvement of treatment adherence [3]. Also, in some cases, complex therapeutic regimens can be hard to implement by the patients due to their health conditions or low level of education, for example.

Non-adherence with the prescribed therapeutic regimen can result in complications for the patient’s health and increase health care costs [4]. It has been shown that a potential solution to reduce hospitalization rates is the education of the patients and their caregivers regarding pathologies and treatments, which contribute to increase adherence to medication and medical recommendations [5,6,7].

Older people represent 6.4% of the world’s population and have become the fastest growing segment of the population, leading to an increase in the prevalence of chronic diseases, such as Alzheimer’s disease, Parkinson’s disease, depression, diabetes, heart disease, and osteoporosis, among others [1]. Living with multiple chronic conditions may negatively affect the patient’s day-to-day life, and the prevalence of cognitive and functional impairments may compromise medication adherence [4]. Additionally, physiological changes due to aging conduce to pharmacokinetics and pharmacodynamics variations, making older patients more vulnerable to adverse effects resulting from non-adherence to therapies [8]. Aging and the growing prevalence of clinical conditions may also affect cognitive abilities, leading to a greater demand for informal and/or formal caregiver support. 

Informal caregivers may be considered primary or secondary caregivers, depending mainly on the time they spend caring for the person, and may live together or separately from the person receiving care [9]. According to the current Portuguese law [10], informal caregivers are defined as “principal” and “non-principal” caregivers. The first may be the spouse, or a relative up to the fourth degree of the person being cared for, who accompanies and cares for the patient on a permanent basis, who lives with them and who does not receive any remuneration for their activity or for the care they provide to the person in need of care. The non-principal informal caregiver monitors and cares for the person on a regular basis, but not permanently, and may or may not receive remuneration for their care of the person. Both have rights and duties, which include receiving training to acquire and develop skills to provide proper care, and benefit from rest periods [10]. Family caregivers are the most important resource for assisting patients with chronic conditions that require long-term care. However, these situations demand a great emotional, physical, and financial cost for informal caregivers, even though they may feel some satisfaction in providing this service. Currently, these caregivers receive little support and assistance, and face various psychological and physical challenges, such as using medical equipment and managing the patient’s medication. 

Patient health outcomes may be affected by medication use, particularly for those who need support to comply with their therapeutic scheme. Polymedication—taking five or more drugs simultaneously—or the prescription of a new drug are two factors that can complicate a patient’s adherence to the prescribed therapy [11]. This also represents a challenge for caregivers, particularly if they are informal caregivers, considering the lack of specifics skills in health and medication management. 

Digital health tools, which include several technologies, may improve patients’ health and provide an adequate care delivery, making a valuable contribution to the prevention and management of non-communicable diseases, and playing a key role in the improvement of adherence to therapy [12].

The integration of electronic health (eHealth) into medication prescription and administration is critical to achieve better outcomes related to medication safety, treatment, and health outcomes [2,13]. These tools can also play a significant role in informing, educating, monitoring, and motivating patients [13].

Within the scope of eHealth, different devices may be used, including mobile devices (mHealth), telecommunications technologies (telehealth), text messages (Short Message Service, SMS), and wearable devices, such as watches or bracelets [13,14]. The term mHealth is defined as the use of cell phones and wireless devices to improve health outcomes, and are a potential solution to increase therapy adherence [15] and education [16]. 

One of the most used devices to improve therapy adherence is the mobile phone due to its portability and constant presence in the patient’s daily routine [17]. Interactive voice response (IVR) is also a potential tool that includes a technology based on the use of mobile phones, allowing the user to interact with the system using a keyboard as an interface [18]. Text messages (SMS) are a short form of communication between mobile phones that are widely used by the general population and constitute another available tool [19]. 

The use of mHealth devices depends on several factors, such as personal motivation and values, connection facilities, and skills related to technology use. The development of these tools requires taking patients’ needs and wishes into account, as well as the possibility that they can also be used by caregivers who can benefit from them, and the possibility of adaptation in cases of low digital literacy or special needs (cognitive or sensory) [12]. In cases of patients with degenerative diseases or some sort of inability to self-medicate, caregivers may be the only users of the mHealth tools [15]. 

Although there are already several digital tools that can be used to increase adherence to therapy, we still need to understand how these tools are being used and what improvements they really bring to the quality of life of patients and/or their caregivers.

The aim of this scoping review is to identify the digital tools currently available for use by informal caregivers that have been shown to have a positive impact on therapy adherence.

## 2. Materials and Methods

This review was carried out by adopting the methodology proposed by Peters et al. [20], and reported according to PRISMA guidelines for scoping reviews [21]. This review’s objective was to assess what IT tools are available for use by informal caregivers and how they may contribute to increasing patients’ medication adherence, improving disease symptoms, and/or improving patients’ quality of life. The research question (Table 1) was defined according to the PICO model [22].

A literature search was carried out in two databases (PubMed and Web of Science) using the keywords and search strategy presented in Supplementary Table S1. These databases were searched from inception to May 2022, and a second search was carried out in January 2023. All references were compiled in an Endnote file and duplicates were removed.

### 2.1. Inclusion and Exclusion Criteria

To be included in this review, the retrieved studies needed to meet the following inclusion criteria: (1) report the use of IT tools by informal caregivers to manage medication for periods longer than 1 week; (2) include patients with a diagnosed chronic disease (for more than 6 months) and their respective caregivers; (3) to be written in English, Spanish, or Portuguese; and (4) report primary studies, with or without a control group, comparing different IT tools, or comparing the use of IT tools with standard methods (without using any technological tools). Articles that report observational studies, revisions, or study cases, the use of IT tools to manage other parameters that do not include medication management or that are related to the management of animal medication, and articles that report the use of IT tools by health professionals or other formal caregivers were excluded from this review.

**Figure 1:**
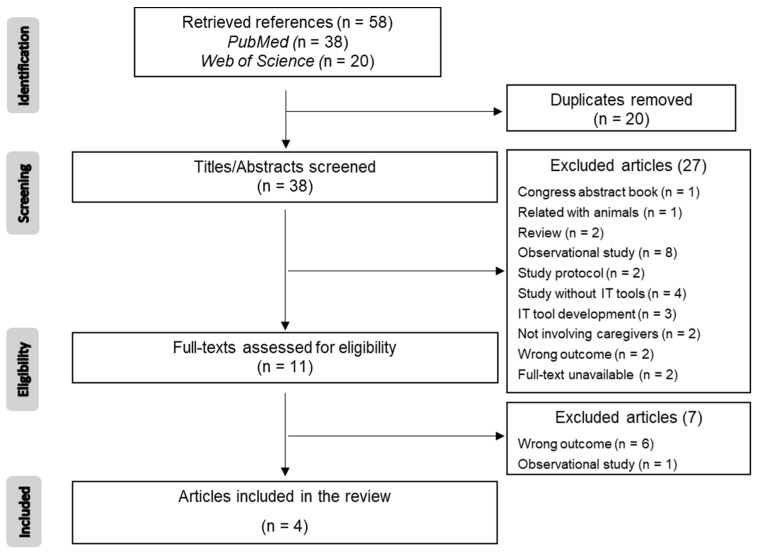
PRISMA flowchart presenting the database searches, the number of titles/abstracts screened, the number of full texts retrieved and analyzed, and the final number of articles included in this review.

Articles were screened independently by all three members of the review team. In the first stage, titles and abstracts were screened to select studies potentially relevant for this review. Afterwards, the full texts of the selected articles were retrieved, and all the reviewers screened them. At the end, only the articles that met the inclusion criteria were included (Figure 1).

### 2.2. Data Extraction

In the next stage, relevant data were extracted and compiled in a specific form prepared for this purpose. Data were organized as follows: (1) study characteristics (authors, title, date of publication, country where the study was carried out, financial support, study design (with or without comparison between intervention and control groups), and patients and caregivers’ eligibility); (2) patients’ and caregivers’ demographic characteristics (age, sex/gender, and race/ethnicity); and (3) intervention description (type of IT tool, duration, role of the caregiver, type of comparison, outcomes, and assessment tools). The data were summarized in tables and presented in a narrative text format.

## 3. Results

The database search yielded 57 articles, 20 of which were duplicated. After the title/abstract screening of the remaining 38 articles, only 11 (27%) full texts were evaluated for eligibility considering the inclusion/exclusion criteria. Most articles were excluded because they reported observational studies (*n* = 8) or they did not included IT tools (*n* = 4). Two full texts were not available, even though the corresponding authors were contacted by the reviewers. The remaining reasons for exclusion are presented in Figure 1. In the end, only four references (10.5% of the total articles identified in the initial search, after deduplication) were included in the present review.

### 3.1. Characteristics of the Studies

Table 2 presents the main characteristics of the studies included in the present review. Three of the four included studies were carried out in the United States and were financed [23,24,25], while the other one was carried out in Japan and does not mention if it was financially supported [26]. Two studies were randomized controlled trials (RCT) [24,25], one was an open label trial [23], and other was a case report [26]. Although all the studies included caregivers, for whom the use of the IT tools under evaluation was intended, only two studies present caregivers’ eligibility criteria [23,24], while all report eligibility criteria for the patients. The IT tool assessed in two studies was the interactive voice response (IVR) system [23,24], while one study used an Automatic Medication Dispenser (AMD) [26], and other used text messages [25]. The studies were carried out for very different periods of time: 3 months [25], 12 months [24], 36 to 54 months, depending on the patient [26], and 3 and 6 months, with two different groups assessed at different time [23].

### 3.2. Interventions

The interventions reported in the included articles enrolled patients with different diseases and demographic characteristics, as well as caregivers with distinct roles during the intervention period. In two studies [23,25], the demographic characteristics of the caregivers were not presented. Additionally, the interventions were also diverse between the studies (Table 3).

The interactive voice response (IVR) technology is a phone system that allows the patient to receive information via prerecorded messages without talking to a person (for example, a health professional). This system was applied and assessed in a study with senior patients (total *n* = 301) with diabetes [23], most of them male (97%) and Caucasian (92.8%). The caregiver’s role was to receive messages with suggestions to better support the patient’s diabetes self-management.

IVR was also applied in an RCT [24], in which a total of 331 senior patients with chronic heart failure (CHF) were enrolled. Most of the enrolled patients were females (99.4%) and white (77%). In this case, the role of the caregiver was to support the patient’s disease self-management. The other included RCT [25] enrolled 25 patients with epilepsy. Most of the participants were Caucasian (92%) and 48% of them were female. Participants were randomly assigned to one of four intervention groups or the control group (which used “The Epilepsy Tool Kit” application created by the National Society for Epilepsy). During the intervention period, the caregivers were to oversee the patients and remind them to take their medication.

The case report presented by Kamimura et al. in 2019 [26] describes the use of an Automatic Medication Dispenser (AMD) by patients suffering from Alzheimer Disease (AD). The system consists of a device in which the medicines are placed, and which can be programmed to alert the patient to take each medicine by means of an alarm and a flashing light. Only four patients (3 males and 1 female), with a mean age of 77.3 ± 4.3 years old, were included in this case report. The caregivers were all female (mean age 55.8 ± 5.3 years old) and their role during the intervention was to fill the devices with the medication and check their patient’s condition. The race/ethnicity of the participants was not reported.

The intervention assessed by Mody et al. [25] included sending text messages to patients and/or their caregivers. Some groups also received a MEMS cap, to add to the pill bottles to allow the automatic recording of the date and time of medication intake, while others received a SimpleMed, which is a smart medication organizer that alerts you when to take medication. Both systems were used to monitor adherence [25].

Medication adherence was the primary outcome assessed in this review. As presented in Table 4, this is a frequently reported issue regarding the treatment of chronic diseases. However, all included studies show that the use of IT tools, such as those tested in these trials, may contribute to increasing medication adherence both in adults and in adolescents with different diagnosed diseases.

The evolution of the disease symptoms and perceived quality of life constitute the secondary outcomes evaluated in this review, but these parameters were not evaluated in two of the studies [25,26]. Aikens et al. [23] reported that significant improvements in physical functioning, depressive symptoms, and diabetes-related stress were associated with time, but no changes were observed in psychological functioning in seniors diagnosed with diabetes. On the contrary, no significant differences in the quality of life were observed over time in patients with heart failure, although the patients in the intervention group presented improvements in several parameters (shortness of breath, weight increase, and in general health, in the cases of more depressed patients).

Although the IT tools under evaluation are meant to be used by informal caregivers to help them to support patients under their care, no specific parameters regarding the satisfaction of caregivers with the systems used (related, for example, to their workload, their capacity to help the patients, or impact on caregiver burden) were reported in the included studies.

## 4. Discussion

This review demonstrates that there are still very few studies carried out that evaluate the benefits of using IT tools to improve medication adherence, or, in a broader sense, to improve disease symptoms and patients’ quality of life. Additionally, the impact of these tools on the role of the informal caregivers has scarcely been evaluated, although, in most cases, the intervention of the caregivers in the process is crucial as they are in constant contact with the patients and, in some cases, they must fill/refill the devices with the medication, positively impacting in medication adherence, as shown, for example, by Triverdi et al. [27]. According to the perspectives of the informal caregivers, this is a role that is become more difficult due to several factors including medication regimen complexity, the relationship with the patients, and the lack of specific tools, training, or information related to the diagnosed disease [28,29]. 

The studies included in the present review were all carried out after participants signed informed consent, but no information is available regarding access to the patients’ private medical records by the caregivers. These studies show that, in some cases, the use of available IT tools may be an asset that can positively contribute to treatment adherence (Table 4). In the case of non-adherence, the procedure depended on the study and the type of intervention being evaluated. For example, Aikens et al. [23] reported that, in this situation, the system responded automatically. Other studies [24,26] do not mention how they dealt with non-adherence situations.

In general, an improvement in adherence to therapy seems to have been achieved using the studied technologies, although the methodologies used to measure this variable are not similar across all studies. The lack of medication adherence direct assessment was already pointed out as an issue to be improved in future studies [26]. A study including home-dwelling older adults with multiple chronic conditions and their informal caregivers has shown their beliefs about polypharmacy. They both expressed their confidence in their health professionals regarding the prescriptions of the medication and, in consequence, several older adults and some informal caregivers revealed that they do not wish to be involved in or informed about their polypharmacy. In contrast, others were willing to control the daily administration of the medication, in order to be certain that the patients they care about adhere to the prescribed regimen. Amongst the other identified beliefs about polypharmacy, it is worth emphasizing that they mentioned that taking several drugs every day becomes an obligation and/or a habit [30]. Long-term adherence is a complex and multifactorial process, in which several variables, such as the type of pathology diagnosed, may influence the outcome. Older people present typically lower adherence rates to medication compared to middle-aged people [31,32]. This phenomenon will probably extend to their caregivers, which means that they will also possibly have a less positive impact on the medication adherence of their family members.

In many cases, depending on the disease and/or their age, patients may require partial or full assistance from caregivers to manage their medication. The problem-solving intervention yielded positive outcomes for both intervention and control groups, as evidenced by improved medication management practices, which may have several explanations: increased attention to medication-management behaviors, increased awareness of caregivers’ medication management, and the medication reconciliation process provided by caregivers in the beginning of the study. In addition, the fact that the included caregivers had high self-efficacy created less room for significant improvements [33]. Similarly, in one of the studies included in this review [25], the medication adherence among the participants was already high, so no proper conclusions could be drawn about the effects of the assessed tool. The results reported by Erlen et al. were obtained with a biased sample of experienced caregivers who volunteered to participate, and it is not possible to conclude if any kind of problem-solving intervention is necessary because the caregivers have already been in their role for several years and have enough experience; however, as observed, the caregivers’ health and burden may contribute to decrease their performance [33]. Automated feedback to caregivers has shown to significantly reduce caregiver burden and improve self-management assistance for patients with chronic heart failure [34]. Also, Roux et al. [35] reported that, after hospital discharge, the daily medication practices, which demand the coordination of medication changes with familiar routines and spaces, as well as an effort from both the patients and their caregivers, may be a challenge to medication adherence. It should also be highlighted that, in many cases, informal caregivers are family members of the patients (spouse/partner, sibling, or adult children) who may also have chronic illnesses and need a medication regimen for themselves [30], which means that they may have their own health and medication adherence compromised by the fact that they need to care for their patient full-time. In this context, the availability of tools that can ease the burden on caregivers may be crucial for the quality of life and health improvement of both the patients and their informal caregivers.

Mickelson and Holden identified several errors and violations related to medication adherence, both intentional and unintentional, and both in occasional and frequent situations, in a study regarding patients diagnosed with heart failure disease and their caregivers [36]. The study showed that there are a set of performance factors that may contribute to these situations, including the availability and quality of tools and technologies to assist patient self-care and medication management. Again, the availability of IT tools that can be used at home to facilitate the patients’ and caregivers’ roles may be an added value to improve, not only the medication adherence, but also the quality of life of both the patients and their informal caregivers, as shown in the studies included in the present review (Table 4). Interventions using different kinds of IT tools (interactive voice response device, automated emails, text messages, or automatic medication dispenser) improved medication adherence regardless of the disease diagnosed (diabetes, chronic heart failure, epilepsy, or Alzheimer’s disease) or the age of the patients enrolled in the studies included in this current review [23,24,25,26]. Additionally, a cross-sectional study enrolling adult patients with type 2 diabetes reported by Mayberry et al. showed that CarePartners (out of home informal support person) may develop a strong relationship with the patients, which seems to favor medication adherence [37]. Mobile devices that facilitate contact between patients and caregivers/CarePartners, allowing them to share doubts and instructions related to medication management, should be considered as part of the support system available for patients at risk of low medication adherence. Patel et al. [38] also reported an improvement in medication adherence for patients suffering from at least one chronic disease and using a smart medication dispenser, connected to the community pharmacy, and a decrease in caregiver burden (both formal and informal caregivers).

### Strengths and Limitations

We can consider that this review has the merit of highlighting the lack of studies evaluating the impact of available IT tools on patients’ adherence to medication and on the burden that informal caregivers can feel when they must provide care for patients who depend on them. In addition, our research has also shown that there is still a lot of work underway to create and implement new automated systems to help patients and caregivers in managing their medication. The development of these digital tools is being carried out in the belief that an intervention for medication management may positively impact on both the health and quality of life of patients, and on the performance and quality of life of informal caregivers. However, these new tools still need to be tested and assessed before becoming available.

Despite the positive contribution of drawing a picture of the current scenario regarding the use of IT tools in medication management, this review has some limitations that should be considered. The number of included studies, the size of the samples in each study, and the diversity of the diseases and age of the patients, as well as the fact that different tools were evaluated in studies with different designs, are factors that do not support generalizing the overall benefits of these tools for informal caregivers and for patients’ adherence to medication.

In the future, studies using adequate designs and enrolling patients and caregivers with well-defined characteristics, suitable for the tools to be tested, are needed to allow the generalization of the effects for each available tool. These studies should include participants with different diseases (in the case of patients) and different age groups, gender, race/ethnicity, level of education, and health literacy (for both patients and informal caregivers). In addition, the scales for assessing the impacts of the tools, such as medication adherence, quality of life, and health outcomes according to clinical condition, should be similar.

## 5. Conclusions

Digital health tools can play a key role in improving patients’ adherence to treatment and self-care, relieving the burden felt by informal caregivers, and ultimately improving the quality of life for both parties involved in the medication management process.

Several digital tools are currently being developed and tested, with the aim of understanding the impact they can have on patients diagnosed with chronic diseases—improving their health outcomes—and their caregivers.

The literature on the impact of these tools in improving adherence to treatment is still scarce. In addition, the studies carried out still include small samples, different adherence assessment methodologies, and, in many cases, do not evaluate the impact these tools have for caregivers.

More well-designed studies that include larger and more diverse samples would allow for a standardized evaluation of the effectiveness of the different developed IT tools in adherence to treatment.

## Figures and Tables

**Table 1:** Definition of the research question, in accordance with the PICO model [22].

**P**	Population	Informal caregivers of patients with chronic diseases
**I**	Intervention	Use of IT tools by informal caregivers
**C**	Comparison	Usual medication management methods, without the use of technology, or use different IT tools
**O**	Outcomes	Medication adherence, disease symptom evolution, and/or quality of life

**Table 2:** Characteristics of the included studies.

Reference	Country	Financial Support	Study Design	Comparison between Groups	Patient Eligibility	Caregivers Eligibility	Duration (Months)	IT Tool
Aikens, et al., 2015 [23]	USA	Yes	Open label trial	No	Reported	Reported	1st wave—3 2nd wave—6	IVR
Piette, et al., 2015 [24]	USA	Yes	RCT	Yes	Reported	Reported	12	IVR
Modi, et al., 2016 [25]	USA	Yes	RCT	Yes	Reported	Not reported	3	Text message
Kamimura, et al., 2019 [26]	Japan	ND	Case report	NA	Reported	Not reported	36 to 54	AMD

NA: not applicable; ND: not determined; AMD: automatic medication dispenser; IVR: interactive voice response; MEMS^®^ 6 TrackCap: Medication Event Monitoring Systems; RCT: randomized controlled trial; SimpleMed+: medication managing and reminder system.

**Table 3:** Characteristics of the interventions and comparison groups reported in the included studies.

							Patients			Caregivers		
Reference	Disease	Intervention	Comparison	Role of the Caregiver	Assessment Tools	Participants (N)	Age (Mean ± SD)	Sex/Gender (%)	Race/Ethnicity (%)	Age (Mean ± SD)	Sex/Gender (%)	Race/Ethnicity (%)
Aikens, et al., 2015 [23]	Diabetes	**Interactive voice response (IVR) calls** to: (a) monitor patients’ symptoms and self-management problems; (b) provide patients with tailored messages about diabetes self-management and medical help-seeking; (c) generate guidance on self-management support for patients’ informal caregivers via structured emails; (d) provide patients’ clinicians with actionable feedback via faxed updates about selected patient-reported health and self-care problems.	NA	Receive emailed summaries of each completed call along with structured suggestions on supporting the patient’s diabetes self-management.	MMAS; SF-12; (PCS and MCS); CES-D; PAID.	1st wave -108 2nd wave -193	66.7 ± 9.8	Female: 3	Caucasian: 92.8	ND	ND	ND
Piette, et al., 2015 [24]	Chronic Heart Failure	**mHealth + CP:** Standard mHealth (as the comparison group) + automated messages (emails) to CP after each IVR call. Messages including feedback about the patient’s status and suggestions for how the CP could support disease care.	**standard mHealth:** weekly IVR calls with questions related to self-management and health. Self-management advice adjusted to patients’ responses, sent to patients; Fax alerts sent to the clinical team when serious health issues were detected.	Support patients’ self-management	Weekly IVR reports; CES-D; MLHFQ; HFSCB; CarePartners feedback at follow-up interview.	331	67.8 ± 10.2	Female: 99.4	White: 77	46.7 ± 13.2	Female: 65	ND
Modi, et al., 2016 [25]	Epilepsy	**Group 1:** Text messages received exclusively by adolescent. **Group 2:** Text message received by adolescent and their caregiver, as well as a single family communication session. **Group 3:** App exclusively for the adolescent. **Group 4:** App for both the adolescent and caregiver, with the single family communication session.	**Group 5 (control group):** The Epilepsy Tool Kit app created by the National Society for Epilepsy.	Oversee/remind to take medication.	MEMS™ SimpleMed+	25	15.7 ± 1.5	Female: 48	Caucasian: 92	ND	ND	ND
Kamimura, et al., 2019 [26]	Alzheimer’s Disease	**Automatic Medication Dispenser (AMD)**	NA	Fill the devices with medications once every 1–2 weeks. Continuously monitor the patients’ conditions (nearly every day).	Medication adherence was calculated by asking the caregivers to count the medications remaining for one week; MMSE; CDR-GS.	4	77.3 ± 4.3	Female: 25	ND	55.8 ± 5.3	Female: 100	ND

NA: not applicable; ND: not determined. APP: application. CDR-GS: Clinical Dementia Rating Global Score. CES-D: Center for Epidemiological Studies Depression Scale. CP: CarePartner. HFSCB: Revised Heart Failure Self-Care Behavior Scale. MEMS™: Medication Event Monitoring Systems TrackCap. MLHFQ: Minnesota Living with Heart Failure Questionnaire. MMAS: Morisky Medication Adherence Scale, for long term medication nonadherence. MMSE: Mini-Mental State Examination. PAID: Problem Areas in Diabetes. SF-12: Medical Outcome Study 12-Item Short Form (PCS: Physical Composite Score and MCS: Mental Composite Score).

**Table 4:** Primary and secondary outcomes reported in the included studies.

Reference	Disease	Primary Outcome: Medication Adherence	Secondary Outcomes: Disease Symptoms Evolution Quality of Life
Aikens, et al., 2015 [23]	Diabetes	Medication nonadherence was the most frequently IVR-reported problem (17.4% of intervention weeks). During intervention, patients became less likely over time to report problems adhering to medication. After intervention, patients showed improvements in long-term medication adherence. Weekly medication nonadherence rate dropped precipitously during the first twelve weeks of intervention, after which it became fairly constant for the remainder of patients’ follow-up.	Time was associated with significant improvements in: - Physical functioning; - Depressive symptoms; - Diabetes-related distress. No significant changes over time regarding psychological functioning. As intervention progressed there were also significant decreases in: - Not performing SMBG; - Not checking feet; - Obtaining SMBG values indicating both high and low blood glucose.
Piette, et al., 2015 [24]	Chronic Heart Failure (HF)	According to 4 HFSCB items (medication adherence), mHealth + CP patients had an 8.8% higher chance of following their prescription exactly at 6 months (62.8% versus 54.0%, *p* = 0.02), and a 13.8% higher chance at 12 months (66.4% versus 52.6%, *p* = 0.01) compared to standard mHealth patients. During the 1-year intervention, patients who received mHealth + CP consistently reported higher levels of perfect medication adherence in the previous week than patients who received standard mHealth.	The HF quality of life (MLHFQ) score and the HF self-care behavior (HFSCB composite score) did not vary by arm at 6 or 12 months. According to patients’ answers, the mHealth + CP arm had more positive and active dyadic communication with their CarePartner. Compared to standard mHealth patients, these patients were much less likely to say they often experience negative emotions when talking with their CP both at 6- and 12-month follow-up. As the follow-up period progressed, mHealth + CP patients, compared to standard mHealth patients: - Were becoming less likely to report shortness of breath during the prior week; - Presented a 4% and 11.1% absolute reduction in the likelihood of reporting shortness at 6 months and 12 months, respectively; - Had a lower probability of having significant weight gains; - Patients with more depressive symptoms at enrollment were more likely to rate their general health as excellent or very good during weekly IVR calls.
Modi, et al., 2016 [25]	Epilepsy	The results support the use of text messaging and application-based reminder systems as acceptable and feasible interventions for adolescents with epilepsy over a short-term treatment plan. The analysis of effect sizes revealed that text messaging may be more effective in enhancing medication adherence compared to application-based interventions, while caregiver involvement did not significantly impact adherence outcomes. Adherence rates exhibited consistency across weekdays and weekends, indicating a stable adherence pattern among adolescents.	Not determined
Kamimura, et al., 2019 [26]	Alzheimer’s Disease	The variability in medication adherence before device use (64% to 100%) was noteworthy, but all participants were consistently supported by their caregivers at nearly every dosing time. This support included prompts to take medication through phone calls or in-person interactions, and even the direct handing of medication to the patient. For 3 patients, medication adherence rate was maintained at 100% (Case D), improved to 100% (Case C), or improved to 95% or above (Case B). The adherence rate of the other patient (Case A) decreased but remained at 79% or above until the follow-up. In addition, all patients demonstrated a strong commitment to medication adherence, rarely requiring reminders or prompts.	Not determined

HFSCB: Revised Heart Failure Self-Care Behavior Scale. IVR: interactive voice response. mHealth + CP: intervention with automated emails sent to the CarePartner after each IVR call. MLHFQ: Minnesota Living with Heart Failure Questionnaire. SMBG: self-monitoring of blood glucose.

## Data Availability

Not applicable.

## Supplementary Materials

The following supporting information can be downloaded at: https://www.mdpi.com/article/10.3390/pharmacy12010020/s1, Table S1: Search strategy—PubMed and Web of Science. In this table, the search strategy used for the PubMed and Web of Science databases is presented.
