# Supplementary material for: Digital Solutions Available to Be Used by Informal Caregivers, Contributing to Medication Adherence: A Scoping Review
Source: Pharmacy (Basel). 2024 Jan 23;12(1):20. doi: 10.3390/pharmacy12010020 (PMC10893508; doi:10.3390/pharmacy12010020)
Supplement: Supplementary file 1 [file pharmacy-12-00020-s001.zip › pharmacy-2772813-supplementary.pdf]

Digital Solutions Available to Be Used by Informal Caregivers, Contributing to Medication Adherence: A Scoping Review

Margarida Espírito-Santo, Sancha Santos and Maria Dulce Estêvão

Table S1. Search strategy – *PubMed* e *Web of Science.*

	Keyword combination
1.	(("medication adherence"[Title/Abstract]) OR ("medication compliance"[Title/Abstract])) OR ("drug adherence"[Title/Abstract])
2.	(((((((("application"[Title/Abstract]) OR ("software application" MeSH[Title/Abstract])) OR ("mobile applications" MeSH[Title/Abstract])) OR ("mobile health"[Title/Abstract])) OR ("mobile health care applications"[Title/Abstract])) OR ("telehealth"[Title/Abstract])) OR ("smartphone apps" MeSH[Title/Abstract])) OR ("technology"[Title/Abstract])) OR ("health information"[Title/Abstract])
3.	(((((("caregiver"[Title/Abstract]) OR ("informal caregivers"[Title/Abstract])) OR ("spouse caregivers"[Title/Abstract])) OR ("family caregiver"[Title/Abstract])) OR ("caretaker"[Title/Abstract])) OR ("informal caretaker"[Title/Abstract])) OR ("patient sitter"[Title/Abstract])
4.	(1) AND (2) AND (3)
